# Very Low Dose Anti-Thymocyte Globulins Versus Basiliximab in Non-Immunized Kidney Transplant Recipients

**DOI:** 10.3389/ti.2023.10816

**Published:** 2023-02-03

**Authors:** Christophe Masset, Clarisse Kerleau, Gilles Blancho, Maryvonne Hourmant, Alexandre Walencik, Simon Ville, Delphine Kervella, Diego Cantarovich, Aurélie Houzet, Magali Giral, Claire Garandeau, Jacques Dantal, the Nantes DIVAT Consortium

**Affiliations:** ^1^ Institut de Transplantation Urologie Néphrologie (ITUN), Service de Néphrologie et Immunologie Clinique, CHU Nantes, Nantes, France; ^2^ INSERM, Center for Research in Transplantation and Translational Immunology, UMR 1064, Nantes Université, Nantes, France; ^3^ Etablissement Francais du Sang, HLA Laboratory, Loos Nantes, France

**Keywords:** diabetes mellitus, rejection, induction therapy, low immunological risk, low dose thymoglobulin

## Abstract

The choice between Basiliximab (BSX) or Anti-Thymocyte Globulin (ATG) as induction therapy in non-immunized kidney transplant recipients remains uncertain. Whilst ATG may allow steroid withdrawal and a decrease in tacrolimus, it also increases infectious complications. We investigated outcomes in non-immunized patients receiving a very low dosage of ATG versus BSX as induction. Study outcomes were patient/graft survival, cumulative probabilities of biopsy proven acute rejection (BPAR), infectious episode including CMV and post-transplant diabetes (PTD). Cox, logistic or linear statistical models were used depending on the studied outcome and models were weighted on propensity scores. 100 patients received ATG (mean total dose of 2.0 mg/kg) and 83 received BSX. Maintenance therapy was comparable. Patient and graft survival did not differ between groups, nor did infectious complications. There was a trend for a higher occurrence of a first BPAR in the BSX group (HR at 1.92; 95%CI: [0.77; 4.78]; *p* = 0.15) with a significantly higher BPAR episodes (17% vs 7.3%, *p* = 0.01). PTD occurrence was significantly higher in the BSX group (HR at 2.44; 95%CI: [1.09; 5.46]; *p* = 0.03). Induction with a very low dose of ATG in non-immunized recipients was safe and associated with a lower rate of BPAR and PTD without increasing infectious complications.

## Graphical Abstract

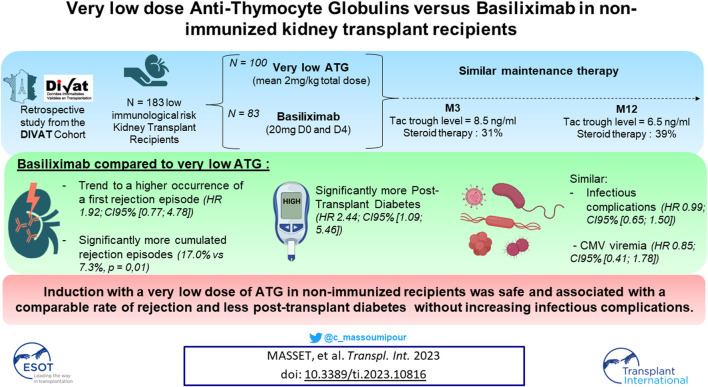

## Introduction

The choice for induction therapy in patients with low immunological risk remains uncertain. Basiliximab (Simulect^®^) is a monoclonal antibody targeting the IL-2 receptor. It results in decreased T cell activation without inducing T cell depletion which has proven beneficial versus placebo controls in induction therapy of kidney transplant recipients (1). Anti-thymocyte Globulins (ATG) consists of polyclonal globulins exerting a strong T cell depleting effect which has proven beneficial against the occurrence of biopsy proven acute rejection (BPAR) in high immunological risk recipients compared to Basiliximab (2). However, this T cell depletion has also been associated with an increased risk of infectious complications, and notably, CMV reactivation (3). Thus, the kidney disease improving global outcomes (KDIGO) guidelines recommends Basiliximab as first line induction therapy in patients with low immunological risk in association with a triple maintenance therapy consisting of calcineurin inhibitors (CNI)—mainly tacrolimus –, antiproliferative drugs (mycophenolate mofetil—MMF; or mycophenolic acid—MPA) and steroids (4). However, ATG remains widely used due to its good protective effect against allograft rejection and other benefits such as a decrease in delayed graft function (5) or the possibility of rapid steroid withdrawal, thus permitting reduction in side effects such as post-transplant diabetes (6, 7). Moreover, ATG’s depleting effect has been proven to depend on the total administered dose, which is currently lower than when first introduced several years ago (8).

In our institution, induction therapy is mainly standardized, even though transplant physicians are free to modify treatment according to the patients’ history. Briefly, induction of non-immunized patients receiving a first kidney transplant consisted mostly of Basiliximab until 2016; and of a very low dose of ATG since 2017. The objective of this study was to retrospectively evaluate the impact of this induction modification on post-transplant outcomes, notably, immunological complications (BPAR and occurrence of *de novo* donor specific antibodies—dnDSA), infectious complications and side effects such as occurrence of post-transplant diabetes (PTD).

## Materials and Methods

### Studied Population

The included patients were adults ≥18 years receiving a first kidney transplantation from heart beating deceased donors, treated with either ATG or Basiliximab (BSX) as induction therapy between 2015 and 2020. Multiple organ transplant recipients were not considered. We only included patients without anti-HLA class I and/or class II antibodies as determined by Luminex^®^ assay (i.e., mean fluorescence index <2000) and without pretransplant DSA determined at a MFI threshold of 1000 at the time of transplantation. The patients in the BSX group received 20 mg of Simulect^®^ (Novartis) intravenously at day 0 and day 4. The patients in the ATG group received 75 mg of thymoglobulin (Sanofi) for 2 days (day 0 and day 1); however, if they weighed 50 kg or less they received only 50 mg of thymoglobulin. All patients received initial corticotherapy during the first days following transplantation (one 500 mg pulse in the BSX group, two 250 mg pulses in the ATG group, followed by oral steroid therapy which was rapidly reduced and withdrawn according to physician’s choice) associated with a maintenance immunosuppressive therapy consisting mainly of tacrolimus with antiproliferative drugs (mycophenolate mofetil or mycophenolic acid). Infectious prophylaxis consisted of trimethoprim + sulfamethoxazole (Bactrim^®^) during at least the first 6 months post transplantation, and until CD4^+^ T cells counts were ≥200/mm^3^, associated with valganciclovir depending on the risk of post-transplant CMV viremia (3 months in case of recipient’s positive serological assay; 6 months in case of recipient’s negative serological assay associated with a transplant from a CMV seropositive donor).

### Available Data

Donor features included allograft status (extended criteria donor or standard criteria donor), donor age, donor sera creatininemia, cause of death, and CMV status. Recipient characteristics included age, gender, BMI, comorbidities (diabetes, hypertension, dyslipidaemia, neoplasia, vascular, cardiovascular history), duration on waiting list, pre-emptive transplantation, and CMV serology status. Transplantation parameters were the cold ischemia time (CIT), use of machine perfusion and the number of HLA-A-B-DR incompatibilities. Patients lost during follow-up were right-censored for mid- or long-term time-to-event. We assumed that the corresponding information were non-informative. For missing data, we voluntarily excluded patients for which the value spread from the initial date was too high (>3 months for 1-year analysis).

### Outcomes

The principal outcome was patient and graft survival, defined by the time between the transplant and the first event requiring return to dialysis, pre-emptive re-transplantation, or death with a functioning graft. Secondly, we studied cumulative probabilities of all infectious complications (bacterial, BkV viremia or BkV nephropathy, CMV viremia or fungal infection), CMV viremia only, occurrence of the first biopsy proven acute rejection episode [BPAR according to the Banff classification (9)], occurrence of post-transplant malignancy and occurrence of post-transplant diabetes (PTD) (for this latter analysis, patients with diabetes before transplantation were excluded).

Protocol biopsies were performed at 3- and 12-month post transplantation. According to KDIGO recommendations, we considered indicated biopsies for suspicion of rejection by the occurrence of one criterion amongst the following: increased creatininemia (>25%) without any explanation, delayed graft function >10 days, occurrence of *de novo* donor specific anti-HLA antibody, new onset of proteinuria, or unexplained proteinuria >3 g per day. Occurrence of *de novo* DSA detected by Luminex^®^ assay, and the eGFR (estimated by MDRD) at 1-year post transplantation were also evaluated (patients that died or were lost during follow-up before the first anniversary were excluded).

### Statistical Analysis

The characteristics at the time of transplantation between ATG and BSX groups were compared using Chi-square tests or Fisher’s exact test for categorical variables and using Student t-tests for continuous variables. To consider possible confounding variables, we weighted the models on the propensity scores (10), which were obtained by a multivariable logistic regression. To consider possible confounders, we weighted the contribution of individuals according to inverse probability (inverse probability weighting - IPW) of the propensity score (PS) (11). The PS was estimated by a multivariable logistic regression with splines on continuous covariates to ensure the log-linearity assumption. If the splines had an OR greater than 10 or less than 0.1 in univariate analysis, the variable was categorized. Stabilized weights were used in order to obtain a pseudo dataset with a similar sample size to the original one and to estimate the average treatment effect in the entire population (ATE) of the exposure (12). The goodness-of-fit of the model was assessed by graphically checking the positivity assumption (Supplementary Figure S1) and studying the standardized differences (Supplementary Tables S1–S7). The adjusted survival curves were obtained using the weighted Kaplan-Meier estimator and compared using the adjusted log-rank test (13). To provide a relative measure of the effect, a Cox model was estimated maximizing the partial weighted likelihood and using a robust estimator for the variance (14). The corresponding hazard proportionality was graphically checked by plotting log-minus-log of the survival function for the variable of interest. Statistical analyses were performed using Plug-Stat^®^ software (www.labcom-risca.com/plug-stat) based on the R software (15). Note, to respect the methodology in causal inference, we did not consider the maintenance therapy in the propensity scores as physicians may adapt the treatment according to the initial induction therapy.

### Ethics Statement

Data were extracted from the French DIVAT cohort (www.divat.fr, approved by the CNIL, n°914184) consisting of recipients monitored in Nantes. The quality of the DIVAT data bank is validated by an annual cross-center audit. All participants gave informed consent. All patients were included and extracted from the DIVAT database, after informed consent. In order to respect confidential medical information, all data were anonymized before analysis.

## Results

### Description of the Cohort

**FIGURE 1:**
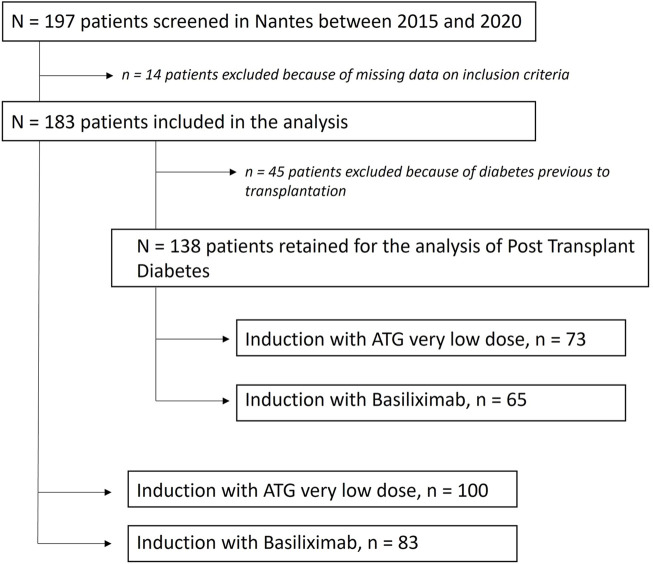
Flow chart of the study.

The study flow-chart is presented in Figure 1. The characteristics of the 183 studied patients at the time of transplantation are presented in Table 1. 83 patients were in the BSX group (45.4%) versus 100 in the ATG group (54.6%). The average total dose of thymoglobulin administered was 2.0 mg/kg, and no serious side effects were noted in this population (notably no serum sickness disease). Of note, 49 patients in the ATG group received ≤2.0 mg/kg of thymoglobulin, while the average dosage of the 51 other patients was 2.3 mg/kg of thymoglobulin.

**TABLE 1:** Description of the entire cohort according to induction therapy.

	Whole sample (*n* = 183)			ATG (*n* = 100)			Basiliximab (*n* = 83)			*p*-value
	NA	n	%	NA	n	%	NA	n	%	
Male recipient	0	131	71.6	0	76	76.0	0	55	66.3	0.1460
Preemptive transplantation	0	33	18.0	0	15	15.0	0	18	21.7	0.2415
Etiology of ESRD										
Glomerulonephritis	0	53	29.0	0	28	28.0	0	25	30.1	0.7529
Tubulo-interstitial (including PKD)	0	60	32.8	0	32	32.0	0	28	33.7	0.8034
Vascular (including Hypertension)	0	34	18.6	0	21	21.0	0	13	15.7	0.3554
Diabetes	0	19	10.4	0	11	11.0	0	8	9.7	0.7637
Undetermined	0	17	9.2	0	8	8.0	0	9	10.8	0.5095
History of diabetes	0	45	24.6	0	27	27.0	0	18	21.7	0.4060
History of vascular disease	0	72	39.3	0	41	41.0	0	31	37.3	0.6148
History of cardiac disease	0	65	35.5	0	43	43.0	0	22	26.5	0.0203
History of cardiovascular disease	0	100	54.6	0	60	60.0	0	40	48.2	0.1102
History of pregnancy	0	37	20.2	0	17	17.0	0	20	24.1	0.2341
History of malignancy	0	45	24.6	0	24	24.0	0	21	25.3	0.8387
History of dyslipidemia	0	114	62.3	0	60	60.0	0	54	65.1	0.4820
Recipient BMI ≥ 25 kg/m^2^	0	92	50.3	0	52	52.0	0	40	48.2	0.6081
Positive recipient CMV serology	1	75	41.2	1	45	45.5	0	30	36.1	0.2038
HLA incompatibilities > 4	0	52	28.4	0	32	32.0	0	20	24.1	0.2379
Use of machine perfusion	0	111	60.6	0	62	62.0	0	49	59.0	0.6516
Male donor	0	108	59.0	0	56	56.0	0	52	62.7	0.3624
ECD donor	0	112	61.2	0	60	60.0	0	52	62.7	0.7141
Vascular cause of death	0	115	62.8	0	63	63.0	0	52	62.7	0.9612
Donor hypertension	5	51	28.7	5	17	17.9	0	34	41.0	0.0007
Positive donor CMV serology	0	87	47.5	0	40	40.0	0	47	56.6	0.0249
	NA	m	SD	NA	m	SD	NA	m	SD	
Recipient age (years)	0	58.2	15.6	0	58.5	16.0	0	57.9	15.1	0.8069
Donor age (years)	1	59.7	17.3	1	60.3	17.0	0	59.0	17.6	0.7992
Donor creatininemia (µmol/L)	0	88.3	51.0	0	89.2	61.3	0	87.3	46.8	0.6285
Duration on waiting list (months)	0	22.0	20.3	0	21.9	21.6	0	22.1	18.6	0.9632
Cold ischemia time (hours)	0	13.3	5.6	0	12.7	5.8	0	13.9	5.1	0.1377

Abbreviations: BMI, body mass index; CMV, cytomegalovirus; EBV, Epstein-Barr virus; ECD, expanded criteria donor; HLA, human leucocyte antigen; NA, not available (missing); sd, standard deviation; ESRD, end stage renal disease; PKD, polycystic kidney disease.

The average recipient age was 58.5 years in the BSX group versus 57.9 years in the ATG group (*p* = 0.80) and a majority of them were male (71.6%). 18% had a preemptive transplantation, 24.6% had history of diabetes and 61.2% received an allograft from an extended criteria donor (ECD) without any significant difference between groups. 41.2% of the recipients had a positive CMV serology (45.5% in the ATG group and 36.1% in the BSX group, *p* = 0.20) and 47.5% of the donors had a positive CMV serology (40.0% in the ATG group and 56.6% in the BSX group, *p* = 0.02).

With respect to steroid maintenance therapy, 31.1% of patients received corticotherapy by month 3 (32.8% in the BSX group and 29.7% in the ATG group, *p* = 0.67) with an average dose of 7 mg/day; and 38.8% at 1 year (38.9% in the BSX group and 38.8% in the ATG group, *p* = 0.16) with an average dose of 7 mg/day. Tacrolimus therapy was the most common immunosuppressant in both groups (95.8% vs. 94.0%, *p* = 0.72 at 3 months and 86.3% vs. 89.4%, *p* = 0.56 at 12 months in the BSX and ATG groups, respectively) with similar trough levels (8.3 ng/mL vs. 8.7 ng/mL at 3 months, *p* = 0.76 and 6.2 ng/mL vs. 6.7 ng/mL at 12 months, *p* = 0.19 in the BSX and ATG groups, respectively). Finally, the use of antiproliferative drugs was similar between patients who underwent an induction by BSX or ATG (respectively 87.6% vs. 92.8% at 3 months, *p* = 0.27 and 78.7% vs. 81.5% at 12 months, *p* = 0.67), Supplementary Figure S2.

### Patient and Graft Survival

**FIGURE 2:**
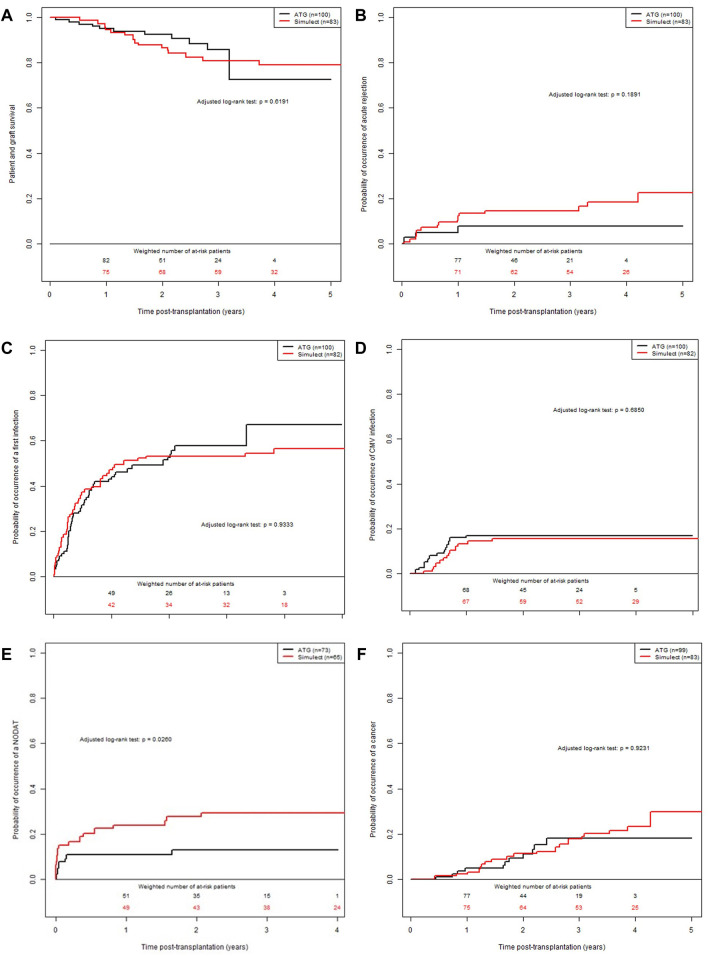
Confounder-adjusted probabilities of events according to the time post-transplantation and the induction therapy. **(A)** patient and graft survival. **(B)** cumulative probability of a first episode of acute rejection episode. **(C)** cumulative probability of infection. **(D)** cumulative probability of CMV replication. **(E)** cumulative probability of PTD. **(F)** cumulative probability of post-transplantation malignancy.

During the follow-up, 18 deaths with a functioning allograft (9 in each group) and 8 returns to dialysis (including 6 in the group BSX) were observed. Median event-free follow-up time was 3.0 years (min: 0.0; max: 5.2). The patient and graft survival, illustrated in Figure 2A, was 95% at 1-year post-transplantation (95% CI: 91%; 99%) in ATG group versus 94% in the BSX group (95% CI: 90%; 100%). These results corresponded to an adjusted HR of 1.22 (95% CI: 0.53; 2.80, *p*-value = 0.63), between patients of the BSX group versus those of the ATG group.

### Occurrence of BPAR Episodes and dnDSA

The cumulative adjusted probabilities of the occurrence of a first BPAR is illustrated in Figure 2B. Median event-free follow-up time was 3.0 years (min: 0.0; max: 5.2). The value for the ATG group was 5% at 1-year post-transplantation (95% CI: 1%; 9%) versus 11% for the BSX group (95% CI: 4%; 17%). These results corresponded to an adjusted HR of 1.92 (95% CI: 0.77; 4.78, *p*-value = 0.15), between BSX patients compared to ATG treated patients. Finally, at 1-year post transplantation, 2 patients in the ATG group and 2 patients in the BSX group presented a DSA with MFI >1000. However, the MFI were rather lower in patients from the ATG group (2147 and 1441) compared to the BSX group (4,900 and 14,000).

During the follow-up, 279 biopsies were performed, 150 in the ATG group and 129 in the BSX group.

Overall, and considering all rejection episodes (first and recurrent), there was significantly more biopsies concluding to a BPAR in the BSX group (*n* = 22, representing 17.0% of performed biopsies) than in the ATG group (*n* = 11, representing 7.3% of performed biopsies), *p* = 0.0152, Supplementary Figure S3.

In the ATG group, BPAR consisted of 7 Borderline lesions (BL) and 4 T Cell Mediated Rejection (TCMR); these later were all successfully treated except for one patient who died with a functional allograft. Of note, among the 11 patients who presented a BPAR in the ATG group, 5 had a total dose < to 2 mg/kg and 6 a total dose > to 2 mg/kg.

In the BSX group, BPAR consisted of 11 BL, 8 TCMR, 1 Antibody Mediated Rejection (ABMR) and 2 mixed rejection (ABMR + TCMR). While 15 BPAR would have require a treatment, only 13 received it (2 patients were considered too frailty). 6 on 13 treated episodes evolved favorably, 6 were refractory and evolved towards end stage renal disease, and one patient deceased with a functional allograft.

### Cumulative Probability of Infectious Complications

During the follow-up, 95 events (including 45 in the group BSX) were observed. Median event-free follow-up time was 3.0 years (min: 0.0; max: 5.0). The cumulative adjusted probabilities of the occurrence of infection is presented in Figure 2C. The value for the ATG group was 43% at 1-year post-transplantation (95% CI: 32%; 52%) versus 47% in the BSX group (95% CI: 35%; 57%). These results corresponded to an adjusted HR of 0.99 (95% CI: 0.65; 1.50, *p*-value = 0.95), between BSX patients versus ATG treated patients.

### Cumulative Probability of CMV Viremia

During the follow-up, 30 events (including 14 in the group BSX) were observed. Median event-free follow-up time was 3.0 years (min: 0.0; max: 5.1). The cumulative adjusted probabilities of the occurrence of CMV viremia is presented in Figure 2D. The value for the ATG group was 17% at 1-year post-transplantation (95% CI: 9%; 24%) versus 13% for the BSX group (95% CI: 6%; 20%). These results corresponded to an adjusted HR of 0.85 (95% CI: 0.41; 1.78, *p*-value = 0.6731), between the two groups of patients.

### Cumulative Probability of Post-Transplant Diabetes

The patient characteristics at the time of transplantation are presented in Supplementary Table S6. Among the 183 patients from the initial cohort, 45 were excluded because they were diabetic before transplantation, leading to a sub-cohort of 138 KTR: 65 patients were treated with BSX (47.1%) and 73 treated with ATG (52.9%). During the follow-up, 27 events were observed (including 18 of the BSX group). Median event-free follow-up time was 3.0 years (min: 0.4; max: 5.1). The cumulative adjusted probabilities of the occurrence of post-transplant diabetes is presented in Figure 2E. The value for the ATG group was 11% at 1-year post-transplantation (95% CI: 3%; 18%) versus 24% in the BSX group (95% CI: 13%; 34%). These results corresponded to an adjusted HR of 2.44 (95% CI: 1.09; 5.46, *p*-value = 0.03), between BSX versus ATG treated patients.

### Cumulative Probability of Post-Transplant Malignancy

During the follow-up, 30 events were observed (19 in the group BSX). Median event-free follow-up time was 3.0 years (min: 0.0; max: 5.2). The cumulative adjusted probability of the occurrence of neoplastic (included squamous cell carcinoma and post-transplant lymphoma disease) is presented in Figure 2F. The value for the ATG group was 0.04 at 1-year post-transplantation (95% CI: 0.00; 0.08) versus 0.02 for the BSX group (95% CI: 0.00; 0.06). These results corresponded to an adjusted HR at 1.04 (95% CI: 0.48; 2.25, *p*-value = 0.9162), between BSX and ATG treated patient groups.

### Evolution of Lymphocyte Count and Allograft Function

**FIGURE 3:**
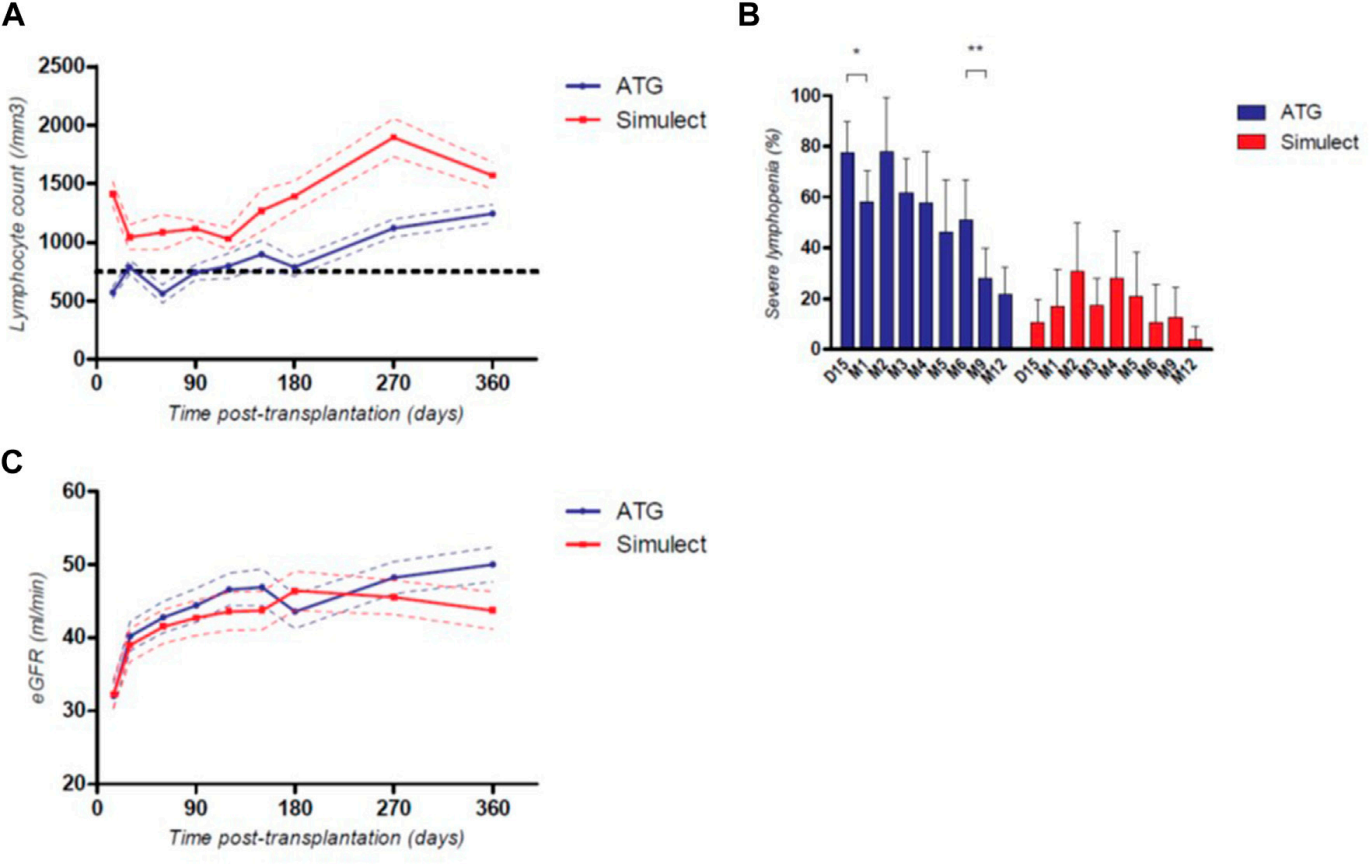
Mean total lymphocyte count **(A)**, percentage of patients with severe lymphopenia (≤750/mm^3^) **(B)** and mean eGFR **(C)** during the first-year post transplantation depending on their induction therapy; *: *p* < 0.05; **: *p* < 0.01; continuous variables are represented with their respective standard mean error, categorical variables are represented with their respective 95CI%.

As expected, total lymphocyte counts were significantly lower during the first-year post-transplantation for patients in the ATG group (Figure 3A). However, after the sixth month post transplantation, the lymphocyte count in these patients increased, and the percentage of patients with severe lymphopenia (<750/mm^3^) decreased significantly (51.1% of patients receiving ATG had a severe lymphopenia at 6-month post transplantation vs. 28.0% at 9-month post transplantation, *p* = 0.01, Figure 3B). In the ATG group, patients who presented a severe lymphopenia at 1-year post-transplantation despite very low doses of thymoglobulin were significantly older (69 years old vs. 53 years old, *p* = 0.0020). Allograft function was globally similar between ATG and BSX groups, however with a trend to a better eGFR at 1-year post transplantation in patients from the ATG group (50.0 vs. 43.7 mL/min, *p* = 0.07), Figure 3C. Of note, there was no significant difference in the occurrence of delayed graft function between groups (26.2% vs. 29.2% in ATG and BSX groups respectively, *p* = 0.74).

## Discussion

Despite multiple studies characterizing induction therapy in low immunological risk kidney transplant recipients, controversies still exist regarding the best treatment to provide for these patients. Basiliximab is currently recommended by the KDIGO guidelines based on studies performed several years ago comparing BSX to ATG (4), notably because of an increased risk of viral infection due to the prolonged T cell depletion induced by ATG. However, as ATG acts in a dose-dependent manner (6), numerous transplant physicians have decreased its dosage over the years in order to reduce its side effects (17, 18).

We demonstrated the consequence of drastically reduced ATG dosage (average of 2 mg/kg total dose) in non-immunized recipients receiving a first kidney transplant, compared to basiliximab-treated patients, undergoing a similar maintenance therapy. Similarly, previous studies on smaller cohorts reported the safety of a very low dose of ATG in low immunological risk patients (19, 20). Other investigated outcomes related to a low dose of ATG, which however remained higher than our and usually associated with long term steroid therapy (21, 22). In relation to immunological complications, we found that a very low dose of ATG seems safe in this low-risk population with a comparable occurrence of a first episode of rejection. Moreover, we even observed a trend in favor of low doses of ATG. This non-significant difference was probably due to an underpowering of the study, and larger series seems mandatory to confirm this later point. Nevertheless, investigation of all BPAR episodes demonstrated a significant lower number of episodes rejection in patients from the very low ATG group. Moreover, patients from the ATG group had a significantly lower occurrence of post-transplant diabetes. One hypothesis is the different number of treated BPAR among groups, as steroids are known to promote post-transplant diabetes. Other reports evidenced a difference in PTD occurrence depending on the induction, but the exact imputability rather than the consecutive maintenance therapy management is still unclear (6, 23, 24). Finally, the small number of studied events may induce a lack of power and validation data from other cohorts will be of interest. Whilst ATG did not significantly impact allograft survival (1, 25), despite a trend to a better 1-year allograft function, it is well known that allograft rejection negatively impacts long-term kidney transplant outcomes (26, 27). Based on our data, a steroid-sparing strategy appears safe in low-immunological risk patients who received a very low dose of Thymoglobulin and can thus be conduce without increasing the risk of allograft rejection.

The use of ATG by transplant physicians is often accompanied by an apprehension of viral infections. In our cohort, according to a well-controlled prophylaxis, there was no significant difference between global infectious complications, nor CMV viremia, using low doses of ATG compared to an induction with Basiliximab. This is concordant with a previous study by our group where we found no difference in infectious complications (notably CMV viremia) regarding ATG administration in elderly kidney transplant recipients (6). Indeed, CMV viremia may be more related to the presence—or not—of the specific CMV cellular immunity rather than the total lymphocyte count (28).

Obviously, the use of ATG resulted in deep lymphopenia in the first months’ post-transplantation. However, the reduced dosage in our cohort led to a higher lymphocyte count at 1-year post transplantation than previously described (29). Moreover, after the sixth month post transplantation, the percentage of patients presenting a severe lymphopenia, which is known to impact patient survival (30), significantly decreased. This shorter time of deep lymphopenia may enhance the use of ATG at a very low dosage in non-immunized kidney transplant recipients, particularly in younger recipients.

Finally, the use of ATG at this very low dose also seemed to permit cost savings for our institution. In France, induction by Simulect^®^ costs around 3,000 euros per patient and 25 mg of thymoglobulin costs around 250 euros. In our cohort, induction by ATG despite basiliximab for non-immunized patients permitted a total saving of 150,000 euros in 5 years (1,500 euros per patient). These results differs from others who found a cost saving using Basiliximab, because the highest dosage of ATG induced more infectious complications (31). However, as we did not perform a cost-effectiveness study assessing all cumulative costs (days of hospitalization, number of consultations, post-transplant complications … ), definitive conclusions are not possible. A recent American cost-effectiveness study revealed that use of ATG appeared to offer cost and outcomes advantages compared to no-induction in kidney transplant recipients (32).

Our study has some limitations, the main one being the relatively small monocentric sample size, with requirement of further validation, ideally based on a randomized clinical trial which remains the gold standard. However, retrospective data from other centers could not have been included as their total ATG dose is considerably higher. Also, based on recent improvements in detecting anti-HLA immunogenicity, determination of low-risk kidney transplant recipients (i.e., total absence of significant anti-HLA antibodies) is now more accurate, and some transplant teams are currently conducting clinical trials to assess the benefit of induction therapy in this low-risk population (33). In our cohort, ATG induced a better prevention of BPAR than Basiliximab, which supports the pursuit of induction therapy for these patients; which however needs to be tailored to provide a very low dose of ATG.

In conclusion, our report highlights that a very low dose of ATG in non-immunized recipients was safe and associated with a lower rate of rejection episodes and post-transplant diabetes, without increasing infectious complications probably because of a reduced duration of deep lymphopenia.

## Nantes DIVAT Consortium

Gilles Blancho, Julien Branchereau, Diego Cantarovich, Anne Cesbron, Agnès Chapelet, Jacques Dantal, Florent Delbos, Clément Deltombe, Anne Devis, Lucile Figueres, Raphael Gaisne, Claire Garandeau, Magali Giral, Caroline Gourraud-Vercel, Maryvonne Hourmant, Christine Kandel-Aznar, Georges Karam, Clarisse Kerleau, Delphine Kervella, Alice Leclech, Claire Leman, Christophe Masset, Aurélie Meurette, Karine Renaudin, Simon Ville, Alexandre Walencik.

## Data Availability

Complete datasets are available upon reasonable request to the corresponding author.

## Abbreviations

95%CI, 95% Confidence Interval; ATG, anti-thymocyte globulins; BMI, body mass index; BPAR, biopsy proven acute rejection; BSX, basiliximab; CIT, cold ischemia time; CMV, cytomegalovirus; CNI, calcineurin inhibitors; DGF, delayed graft function; DSA, donor-specific antibodies; ECD, expanded criteria donor; eGFR, estimated glomerular filtration rate; HLA, human leucocyte antigen; HR, hazard ratio; KDIGO, kidney disease improving global outcomes; MMF, mycophenolate mofetil; MPA, mycophenolic acid; OR, odds-ratio; SCr, serum creatinine; sd, standard deviation; RMST, restricted mean survival time; RMTL, restricted mean time lost.
