# Supplementary material for: Very Low Dose Anti-Thymocyte Globulins Versus Basiliximab in Non-Immunized Kidney Transplant Recipients
Source: Transpl Int. 2023 Feb 3;36:10816. doi: 10.3389/ti.2023.10816 (PMC9935561; doi:10.3389/ti.2023.10816)
Supplement: Supplementary file 1 [file DataSheet1.docx]

**Supplementary material**

**Table S1**. The pseudo-sample after weighting on the propensity scores for the analysis related to the patient and graft survival (abbreviations: ATG, anti-thymocyte globulin; BSX, basiliximab)

|  | **Whole sample** | | **ATG** | | **Simulect** | | **Standardized** |
| --- | --- | --- | --- | --- | --- | --- | --- |
|  | **n** | **%** | **n** | **%** | **n** | **%** | **Difference (%)** |
| **History of diabetes** | 46.8 | 25.6 | 25.4 | 25.5 | 21.4 | 25.8 | 0.6 |
| **History of cardiovascular disease** | 100.6 | 55.0 | 54.9 | 55.1 | 45.7 | 54.9 | 0.4 |
| **ECD donor** | 110.4 | 60.4 | 60.3 | 60.5 | 50.1 | 60.2 | 0.7 |
|  | **m** | **SD** | **m** | **SD** | **m** | **SD** |  |
| **Recipient age (years)** | 58.1 | 15.6 | 58.2 | 15.6 | 58.1 | 15.6 | 0.5 |
| m, mean; SD, standard deviation. | | | | | | | |

**Table S2**. The pseudo-sample after weighting on the propensity scores for the analysis related to time to the first infection (abbreviations: ATG, anti-thymocyte globulin; BSX, basiliximab)

|  | **Whole sample** | | **ATG** | | **Simulect** | | **Standardized** |
| --- | --- | --- | --- | --- | --- | --- | --- |
|  | **n** | **%** | **n** | **%** | **n** | **%** | **difference (%)** |
| **History of hypertension** | 165.9 | 90.8 | 91.1 | 90.9 | 74.8 | 90.8 | 0.5 |
| **Positive donor CMV serology** | 87.5 | 47.9 | 48.0 | 47.9 | 39.5 | 48.0 | 0.2 |
|  | **m** | **SD** | **m** | **SD** | **m** | **SD** |  |
| **Recipient age (years)** | 58.8 | 15.3 | 58.7 | 15.3 | 58.9 | 15.4 | 1.0 |
| **Duration on waiting list (months)** | 22.6 | 20.8 | 22.3 | 20.7 | 22.9 | 21.0 | 2.8 |
| m, mean; SD, standard deviation. | | | | | | | |

**Table S3**. The pseudo-sample after weighting on the propensity scores for the analysis related to time to the first CMV infection (abbreviations: ATG, anti-thymocyte globulin; BSX, basiliximab; CMV, cytomegalovirus)

|  | **Whole sample** | | **ATG** | | **Simulect** | | **Standardized** |
| --- | --- | --- | --- | --- | --- | --- | --- |
|  | **n** | **%** | **n** | **%** | **n** | **%** | **difference (%)** |
| **History of malignancy** | 43.2 | 23.7 | 23.7 | 23.8 | 19.4 | 23.7 | 0.2 |
| **Positive recipient CMV serology** | 72.0 | 39.6 | 39.9 | 40.0 | 32.1 | 39.1 | 1.8 |
| **HLA-A-B-DR incompatibilities > 4** | 50.9 | 28.0 | 28.1 | 28.1 | 22.8 | 27.8 | 0.7 |
| **Recipient age ≥60 years** | 95.6 | 52.6 | 52.7 | 52.8 | 42.9 | 52.2 | 1.3 |
| **Cold ischemia time ≥12 hours** | 96.5 | 53.1 | 52.8 | 52.9 | 43.7 | 53.2 | 0.6 |
|  | **m** | **SD** | **m** | **SD** | **m** | **SD** |  |
| **Donor age (years)** | 59.3 | 17.7 | 59.4 | 17.8 | 59.2 | 17.6 | 0.9 |
| m, mean; SD, standard deviation. | | | | | | | |

**Table S4**. The pseudo-sample after weighting on the propensity scores for the analysis related to time to the first acute rejection episode (abbreviations: ATG, anti-thymocyte globulin; BSX, basiliximab)

|  | **Whole sample** | | **ATG** | | **Simulect** | | **Standardized** |
| --- | --- | --- | --- | --- | --- | --- | --- |
|  | **n** | **%** | **n** | **%** | **n** | **%** | **difference (%)** |
| **Recipient blood group** |  |  |  |  |  |  | 1.7 |
| **A** | 89.2 | 48.8 | 48.8 | 48.8 | 40.3 | 48.8 |  |
| **AB** | 10.3 | 5.6 | 5.5 | 5.5 | 4.8 | 5.8 |  |
| **B** | 16.1 | 8.8 | 8.9 | 8.9 | 7.1 | 8.6 |  |
| **O** | 67.3 | 36.8 | 36.8 | 36.8 | 30.5 | 36.9 |  |
|  | **m** | **SD** | **m** | **SD** | **m** | **SD** |  |
| **Cold ischemia time (hours)** | 13.3 | 5.5 | 13.3 | 5.5 | 13.3 | 5.5 | 0.4 |
| m, mean; SD, standard deviation. | | | | | | | |

**Table S5**. The pseudo-sample after weighting on the propensity scores for the analysis related to time to PTD (abbreviations: ATG, anti-thymocyte globulins; BSX, basiliximab)

|  | **Whole sample** | | **ATG** | | **Simulect** | | **Standardized** |
| --- | --- | --- | --- | --- | --- | --- | --- |
|  | **n** | **%** | **n** | **%** | **n** | **%** | **difference (%)** |
| **ECD donor** | 73.6 | 53.5 | 38.8 | 53.3 | 34.8 | 53.7 | 0.8 |
| **Recipient BMI ≥24.5 kg.m-2** | 72.2 | 52.5 | 38.4 | 52.8 | 33.8 | 52.3 | 1.0 |
|  | **m** | **SD** | **m** | **SD** | **m** | **SD** |  |
| **Recipient age (years)** | 55.5 | 16.0 | 55.6 | 16.1 | 55.3 | 16.0 | 1.6 |

**Table S6.** Description of the study cohort for post-transplant diabetes occurrence according to induction therapy (abbreviations: BMI, body mass index; CMV, cytomegalovirus; EBV, Epstein-Barr virus; ECD, expanded criteria donor; HLA, human leucocyte antigen; NA, not available (missing).

|  | **Whole sample (n=138)** | | | **ATG (n=73)** | | | **Simulect (n=65)** | | | **p-value** |
| --- | --- | --- | --- | --- | --- | --- | --- | --- | --- | --- |
|  | **NA** | **n** | **%** | **NA** | **n** | **%** | **NA** | **n** | **%** |  |
| **Male recipient** | 0 | 93 | 67.4 | 0 | 53 | 72.6 | 0 | 40 | 61.5 | 0.1664 |
| **Preemptive transplantation** | 0 | 26 | 18.8 | 0 | 10 | 13.7 | 0 | 16 | 24.6 | 0.1016 |
| **History of vascular disease** | 0 | 47 | 34.1 | 0 | 25 | 34.2 | 0 | 22 | 33.8 | 0.9605 |
| **History of cardiac disease** | 0 | 40 | 29.0 | 0 | 29 | 39.7 | 0 | 11 | 16.9 | 0.0032 |
| **History of cardiovascular disease** | 0 | 65 | 47.1 | 0 | 39 | 53.4 | 0 | 26 | 40.0 | 0.1148 |
| **History of malignancy** | 0 | 33 | 23.9 | 0 | 18 | 24.7 | 0 | 15 | 23.1 | 0.8280 |
| **History of dyslipidemia** | 0 | 80 | 58.0 | 0 | 40 | 54.8 | 0 | 40 | 61.5 | 0.4230 |
| **Positive recipient CMV serology** | 1 | 52 | 38.0 | 1 | 33 | 45.8 | 0 | 19 | 29.2 | 0.0455 |
| **Male donor** | 0 | 80 | 58.0 | 0 | 43 | 58.9 | 0 | 37 | 56.9 | 0.8139 |
| **ECD donor** | 0 | 74 | 53.6 | 0 | 36 | 49.3 | 0 | 38 | 58.5 | 0.2822 |
| **Vascular cause of death** | 0 | 82 | 59.4 | 0 | 42 | 57.5 | 0 | 40 | 61.5 | 0.6325 |
| **Donor hypertension** | 5 | 31 | 23.3 | 5 | 8 | 11.8 | 0 | 23 | 35.4 | 0.0013 |
| **Positive donor CMV serology** | 0 | 65 | 47.1 | 0 | 29 | 39.7 | 0 | 36 | 55.4 | 0.0658 |
| **HLA incompatibilities > 4** | 0 | 33 | 23.9 | 0 | 20 | 27.4 | 0 | 13 | 20.0 | 0.3092 |
| **Recipient BMI ≥ 25 kg/m^2^** | 0 | 72 | 52.2 | 0 | 41 | 56.2 | 0 | 31 | 47.7 | 0.3200 |
|  | **NA** | **m** | **SD** | **NA** | **m** | **SD** | **NA** | **m** | **SD** |  |
| **Recipient age (years)** | 0 | 55.6 | 16.0 | 0 | 55.9 | 16.6 | 0 | 55.2 | 15.4 | 0.8094 |
| **Donor age (years)** | 0 | 57.0 | 17.7 | 0 | 57.2 | 17.9 | 0 | 56.8 | 17.7 | 0.8788 |
| **Duration on waiting list (months)** | 0 | 22.0 | 20.1 | 0 | 20.6 | 21.0 | 0 | 23.6 | 19.1 | 0.3881 |
| **Cold ischemia time (hours)** | 0 | 13.0 | 5.4 | 0 | 12.5 | 5.9 | 0 | 13.7 | 4.7 | 0.1856 |

**Table S7**. The pseudo-sample after weighting on the propensity scores for the analysis related to time to post-transplantation malignancy (abbreviations: ATG, anti-thymocyte globulins; BSX, basiliximab)

|  | **Whole sample** | | **ATG** | | **Simulect** | | **Standardized** |
| --- | --- | --- | --- | --- | --- | --- | --- |
|  | **n** | **%** | **n** | **%** | **n** | **%** | **difference (%)** |
| **History of diabetes** | 43.7 | 24.0 | 24.0 | 24.2 | 19.8 | 23.9 | 0.6 |
| **History of cardiovascular disease** | 99.9 | 55.0 | 54.9 | 55.3 | 45.1 | 54.5 | 1.7 |
| **History of malignancy** | 42.7 | 23.5 | 23.0 | 23.1 | 19.7 | 23.9 | 1.7 |
| **Positive recipient CMV serology** | 72.5 | 39.9 | 39.7 | 40.0 | 32.8 | 39.7 | 0.7 |
| **Positive recipient EBV serology** | 176.1 | 96.8 | 96.0 | 96.8 | 80.1 | 96.9 | 0.9 |
| **Male donor** | 109.6 | 60.3 | 59.7 | 60.2 | 49.9 | 60.4 | 0.3 |
| **ECD donor** | 108.5 | 59.7 | 59.4 | 59.9 | 49.2 | 59.5 | 0.8 |
| **Recipient age ≥60 years** | 94.5 | 52.0 | 51.6 | 52.1 | 42.9 | 51.9 | 0.4 |
| **Recipient BMI ≥25.3 kg.m-2** | 90.3 | 49.7 | 49.4 | 49.8 | 41.0 | 49.6 | 0.4 |
| **Cold ischemia time ≥12 hours** | 95.5 | 52.5 | 52.0 | 52.5 | 43.5 | 52.6 | 0.2 |
| m, mean; SD, standard deviation. | | | | | | | |

|  | 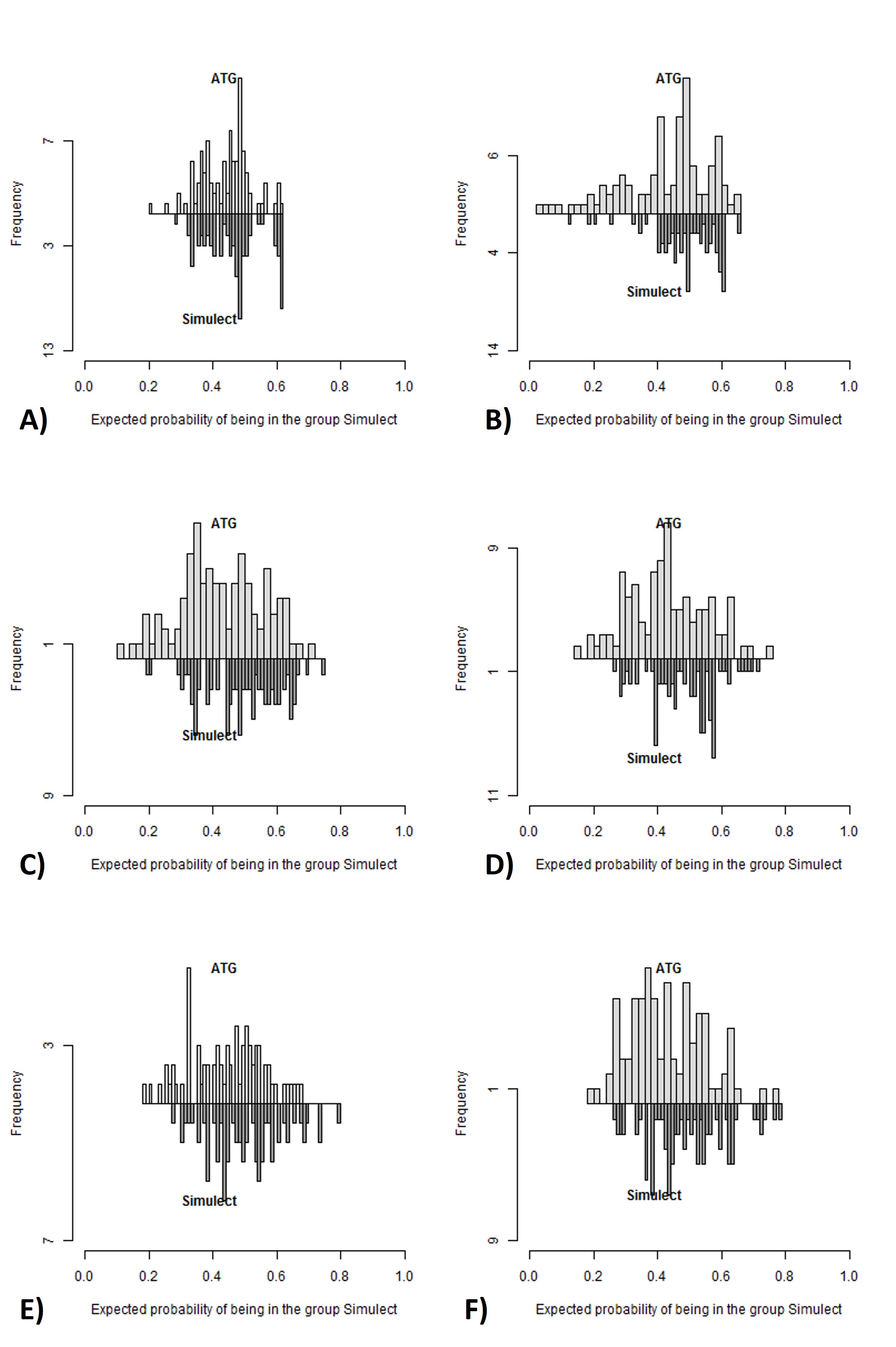  Figure S1. Propensity score distribution. A-patient and graft survival. B-time to first rejection. C-time to first infection D- time to CMV infection E- time to post transplant diabetes. F – time to post-transplant cancer |
| --- | --- |

Figure S2. Percentage of patients receiving tacrolimus therapy (A) and their corresponding average trough levels (B) during the first-year post transplantation depending on their induction therapy. Percentage of patients receiving antiproliferative drugs (C) and steroids (D) as maintenance therapy during the first year-post transplantation depending on their induction therapy.


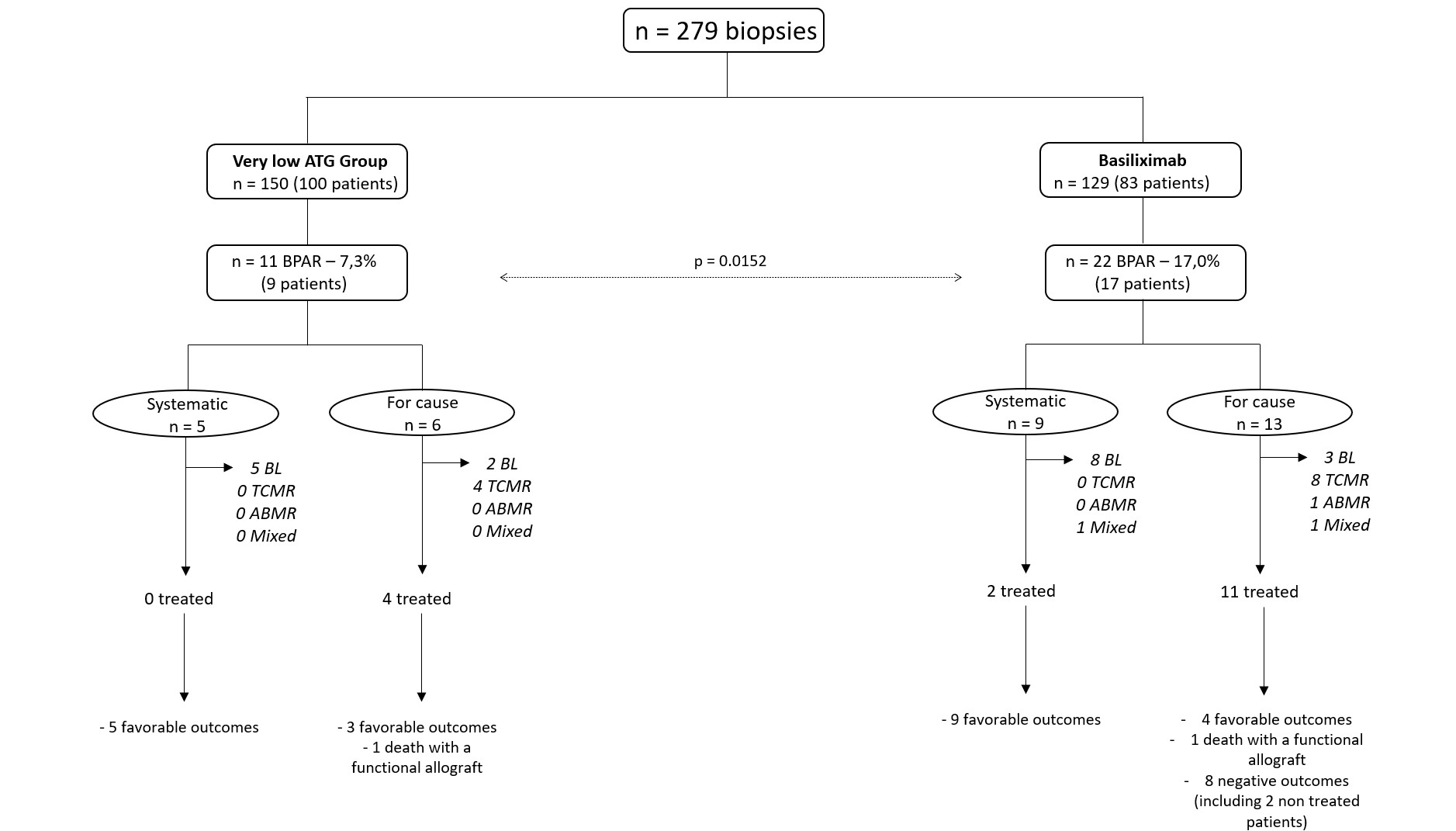


Figure S3. Description of all Biopsies Proven Acute Rejections (BPAR) and their evolution in both groups.

*BL refers to Borderline rejection, TCMR to T Cell Mediated Rejection, ABMR to Antibody Mediated Rejection and Mixed to the simultaneous occurrence of TCMR and ABMR. All BPAR were categorized following the international Banff classification*
